# Atlanta Medical College

**Published:** 1857-05

**Authors:** 


### EDITORIAL AND MISCELLANEOUS.

### ATLANTA MEDICAL COLLEGE.

Without going into all the facts and circumstances connected with the relations which have existed between the Atlanta Medical College and the Medical Department of the University of Nashville, but rather incidentally in reference to the course of those controlling that department, we remarked in the March number of this Journal, that in our opinion, they had pursued an illiberal and ungenerous course, and had manifested an arrogant and selfish spirit, and though we talked thus plainly, as it was our purpose to do (feeling that self-respect and a proper regard for our rights not only justified, but required it,) we intimated a disposition to indulge a forgiving spirit, and even signified our willingness to admit of the Nashville School, all they have been claiming for themselves in the modest, elegant and pious style of "before God and the Saints, the University of Nashville had, *by far*, a larger class of bona fide paying students than any College, outside of Philadelphia, in the United States."

Our opinion of the controlling influence in the Nashville School being such as we have stated, we felt that the pursuit of an open and manly course made it proper for us to make the declaration, that we were not dependent upon the countenance and favor of that Institution, which had been so constantly and industriously held up, by the enemies of this school, as the insuperable obstacle in the way of our success.

The opinion entertained and expressed in reference to the Nashville School, has not been altered by the elaborate and offensive article contained in the last number of the Nashville Journal, doubtless designed as the opening gun to what they intend as the destructive fire which is to be poured upon us at the approaching meeting of the American Medical Association.

We have no taste for controversy, and have not "tossed the glove defiantly," or otherwise; but have thought proper to state openly and candidly what our impressions have been in reference to the conduct and bearing of the Nashville Faculty, under the influence of a spirit, as we believe, whose motto is, "rule or ruin," and while we have at last had an open declaration of that war which we know to have been carried on against us, in a different manner, we here state, that it is our determination to be just to the Nashville School, without regard to the course she may think proper to pursue.

In proof of our disposition to accord full justice to the Nashville School, we intimated in the very article which has been made the occasion of the recent attack upon us, that it was possible, that she might realize the heighth of her apparent ambition, in becoming "the leading School (so far, at least, as the number of students in attendance can make it so) in the United States;" and stated distinctly, that "we had no objection to the high destiny which seemed to await the University of Nashville," and, in addition, that "it was an Institution, taken altogether, to which we looked with pride and pleasure;" in justification of which statements, we will adopt the language of the Nashville Journal, that "there is nothing inconsistent in admiring a man, without endorsing all his habits." It is true, that the Nashville Journal states that they have not been ungenerous to the Atlanta Medical College, and that they have not manifested an arrogant and selfish spirit; and in proof of which, present the fact, that *so far* from the College of Nashville complaining because the Atlanta Medical College chose to hold her sessions in the summer,* "*that with the full permission of all his colleagues, whom he consulted, a Professor here* (there) *assisted Atlanta in her first course*," and asks the question, "was this ungenerous?"

In response to this direct enquiry, we admit the fact, that *Professor Buchanan was allowed by the Nashville Faculty to be a party to the establishment of the Atlanta Medical College*, and thus committed themselves to the acknowledgment, that the Institution was based upon principles equally high and honorable with their own, which we state to be the fact, and can abundantly sustain.

---

* Italics are ours.

In addition to this, we state the fact, that Professor Buchanan was regularly elected, by the Board of Trustees, to the Chair of Anatomy, and early in the course, his name was *published* as holding that relation; and farther, that every member of the Board of Trustees, and the Faculty, considered him a Professor, fully entitled to all the privileges connected with that position, and expected him to discharge all the duties connected with that office; that Professor Buchanan considered himself as occupying this relation to the Atlanta Medical College, with the full permission of his colleagues, and as being under obligations to do all that any other member of the faculty was expected to perform, even to the signing of the diplomas, is clear and unquestionable, and will be fully admitted by him.

"So far," the Nashville School have not been ungenerous or illiberal; "so far" from this, a tender and fostering care had been exercised over the welfare of the little bantling (or "little sister," as they are pleased to term it,) and we are not certain that some did not even suppose that they intended it as a sort of *appendage* to the Nashville School, so remarkable, we may say, unprecedented, had been the generosity, and almost *parental* regard manifested by that Institution. But, to proceed: a large class assembled, and we will merely suggest the *possibility* (in the absence of any other sufficient explanation) that the idea of *competition* may have originated in some *visionary's* brain, (it could not have arisen elsewhere,) and have been whispered in the ear of Nashville, (at least, they intimate in the article alluded to, that we thought they were jealous, and as we have made no such charge, the fair inference is, that they have *suspected* themselves of entertaining such a feeling,) after which, whether, as a result or not, we will leave others to determine, we hear mutterings of disaffection, and then a protest against the propriety of the publication, made long before, of Professor Buchanan's name as Professor of Anatomy, accompanied with an order directed to him to sever his connection with the Atlanta Medical College, under penalty, upon refusal, of losing his position in Nashville.

Fortunately, for Professor Buchanan, (upon whom we are not disposed to cast any censure in that matter, having rendered us efficient service, and, at all times, endorsed in the fullest manner, the correctness of the principles upon which the Institution was established,) the Secretary of the Board of Trustees had neglected to notify him of his election to the Chair of Anatomy, and formally ask his acceptance, and thus he was permitted to escape from the most unenviable position in which he had been placed by his colleagues.

What was the respective influence of each member of the faculty of Nashville, in the adoption of the course, with which we have dealt so mildly, in characterizing it as illiberal and ungenerous, we do not pretend to know; we can only say, that the impression, at that time, was, and still exists, that personal considerations upon the part of the individual who is understood to have the controlling influence in the Nashville School, and who was known to be inimical to Professor Buchanan, had largely shared: let this be as it may, without going into farther detail, we ask the question, if the facts here presented imposed a debt of gratitude, as intimated, upon the Atlanta Medical College towards the Medical Department of the University of Nashville; or, upon the other hand, are we not fully sustained in the opinion which we have thought proper to express of the course pursued toward us?

"But, Atlanta chose to take the initiative in another untried direction:" to this, we reply, that it does not accord with the facts of history, as the University of Virginia, many years since, established the principle of allowing students to come forward for graduation, without *any* interval in the course of instruction; and this Institution, with not more than three or four, certainly not a full corps of Professors, has been fully recognized by the highest Colleges in the land; and was even commended for making *attainments*, not *time*, a test of the qualifications of the candidate for a degree, by a Medical Journal, of which the great Drake was one of the Editors. We contend, that our regulations, even in regard to the length of *time* which a student shall occupy in the study of medicine, before becoming a candidate for graduation, are the same with Nashville, and *challenge* them to the proof, that we do not adhere as strictly to the requisition as *themselves*.

While we hesitate not to say, that we are not of those who are constantly heralding forth the inferiority of the American Medical profession, we freely admit, that there are defects in the system of medical education, and have long since announced ourselves as prepared to confer upon this subject, and to aid in elevating the standard of professional qualification; we say *confer*, and mean to be understood. We intend that those who control the Nashville School shall understand that our claim to a voice in the regulation of medical matters in this country is equal to their own, either as derived from State Sovereignty, or according to the terms of admission into the American Medical Association, with which they have seen fit to threaten us, in reference to their successful control of which, they seem to have forgotten, there may be some doubt; though it is true, that it would seem to be a pity, that there should have been so large an expenditure of time and money, both last year and this, upon the part of our *regulators*, if they should not accomplish our ruin through the instrumentality of this body. That the American Medical Association has no control over this subject, and that, in the very nature of things, it is an *absurdity* to contend for the power of this body to regulate medical education, can be and will be clearly demonstrated, upon the proper occasion; besides this, the rights and interests of all, who are, by their own regulations, entitled to representation in that body, must be respected, even within the limits of their own legitimate sphere of action, and therefore we contend that they have no right to *legislate out* of their body those already belonging to it, or entitled to representation in it--that "the Association is not a law making power for the profession at large," is too plain to admit of question, and "having a code of ethics for the government of *its members*, all the power it has is to cut them off from membership for non-conformity to its code. This it has done, and this, we trust, it will always do, whenever occasion requires."* Having laid down their code of ethics—the adoption of which and conformity to which, entitles all its members to equal rights and privileges in that body—any new exactions not specified in that code would be ex post facto in their claims and entitled to no respect.

In reference to the last point, which we propose to notice at this time, in the article contained in the organ of the Nash-

* S. W. Butler, M. D., Editor of New Jersey Med. and Surg. Reporter, and Chairman of Standing Committee of American Medical Association on Medical Education.

ville School, we have to say, that it seems to be a little remarkable, without having, as they state, *asked any one*, they should be able to speak so confidently as to our standing with other institutions; in reference to that statement, we must be permitted to say, that it is calculated to convey a *false* impression, in regard to the University of Pennsylvania, the only one of the three Colleges mentioned, with whose position we have made ourselves acquainted, our special interest in reference to the relation we sustained to that institution, having very naturally arisen from the fact that it was our Alma Mater.

What the position of the Nashville School is in regard to the Atlanta Medical College, we do not know, as she has never given it officially, so far, at least, as our information extends, but if it is intended, by the statement in the Nashville Journal, to convey the impression (which seems to be the object,) that the *degree* of this College is not recognized as *equal* to their own, then we say, that the assertion, that they stand in precisely the same relation to us, with that of the University of Pennsylvania, is not in accordance with the testimony, and which we stand prepared to prove whenever it is questioned.

In addition, we do not hesitate to say that the statement of the Nashville Organ, that "they are doing precisely what the first Colleges in the United States are doing," is not in accordance with the facts, if it is intended to mean, that the first Colleges in the United States do not *fully recognize* the Atlanta College, and put her upon the same footing with the Medical Department of the University of Nashville, in proof of which, we present the fact, that the *leading* School of the Union, the Jefferson Medical College, of Philadelphia, has, in the most satisfactory and unequivocal manner, time and again, acknowledged the Atlanta Medical College to the fullest extent that we could possibly desire—that the *leading* school in our own State, and certainly *one* of the first Colleges in the United States—the Medical College of Georgia—has admitted our claims as fully equal to her own, and stands most intimately connected with, and committed to her policy, as equally high and honorable with her own—that the Medical College of the State of South Carolina, in Charleston, a long established and most successful Institution, and maintaining a high standard of qualification for graduation, even in these lax days, and located in the very land of honor, does not fail in the most decided manner, to extend to us the cordial grasp of full recognition, and without going into farther detail, we would remark, that if any student of medicine has ever been refused permission to become a candidate for graduation in *any* College of the United States, after attending but a *single* course of lectures, and that in the Atlanta Medical College, we are yet to hear the statement. But without adding more to an exposition of facts, which we have been compelled to make in defending ourselves from the unjust attack, which has been made upon us by the Nashville School, just time enough for general circulation before the opening of the next course of Lectures, and just upon the eve of the meeting of a medical body, with which they have sought to injure us; and hold up in terror before us—but as it was supposed, not time enough to allow a reply through a regular medium, we take leave, for the present, of the Nashville Organ, with the endorsement of its remark, that "there *is* no accounting for taste," as will be clearly demonstrated upon some future occasion, if necessary, from the refined and elevated effusions of a "Western Editor."

